# Communications

**Published:** 1867-05

**Authors:** 


LOUISVILLE, KY., *March 7th*, 1867.

DEAR REGISTER:—

At the semi-annual meeting of the Central States' Dental Association, last year, discussion turned upon the subject of dentrifices, though not in regular debate. Many of the members having frequently been asked by their patients whether they endorsed the use of "Sozodont?" (that being the most popularly *advertised* preparation for the teeth.)

In order to be able to pronounce intelligently in reference to this matter, it was decided to have "Sozodont" analyzed. I herewith transmit the Chemist's report, together with a *quantity* of soap, obtained from the liquid "Sozodont."

For the benefit of the readers of the REGISTER, you are at liberty to publish the communication.

Yours truly,

J. A. McCLELLAND.

